# The Nature of General Shields’ Wound

**Published:** 1848-03

**Authors:** 


*The nature of* Gen. Shields' *Wound.*—This gallant soldier has recently been the guest of our city, and we were called upon to dress his second wound: being detained, we found our friend, Dr. Dugas, in attendance when we arrived. It is known that Gen. Shields was wounded twice in the recent battles in Mexico. By the discharge of a *cannon* at Cerro Gordo, he was shot through the body and given over as certain to die. The General thinks it was a grape shot that traversed his chest. The ball has evidently passed between the *lungs, through the mediastina;* entering within the right nipple and passing out near the spine on the right side. He spate no blood, did not fall, and even gave the word of command after being wounded. In a few moments he was in indescribable agony, and prayed even for death, to be relieved.

None but a medical man can fully apprecite the nature of this wound, which has no parallel on record.—*Southern Med. and Surg. Journal.*

